# THE DUKE INTERAGENCY CARE TEAM: A BRIDGE TO GERIATRIC COMMUNITY RESOURCES

**DOI:** 10.1093/geroni/igz038.943

**Published:** 2019-11-08

**Authors:** Geraldine E Kanne, Melissa Black, Marilyn Disco, Rhonda Mack-Minniefield, David Halpern, Gina Upchurch, Heidi White, Mitchell T Heflin

**Affiliations:** 1 Duke University Health System, Durham, North Carolina, United States; 2 Triangle J Area Agency on Aging, Durham, North Carolina, United States; 3 Senior PharmAssist, Durham, North Carolina, United States; 4 Durham Medical Center, Durham, North Carolina, United States; 5 Duke University Medical Center, Durham, North Carolina, United States

## Abstract

The Duke Geriatric Workforce Enhancement Program aims to improve linkages between primary care practices (PCP’s) and community-based organizations by developing an interdisciplinary, community-based team to consult with PCPs, identifying resources to help vulnerable older adults. The Inter-agency Care Team (ICT) includes a nurse practitioner, pharmacists, community resource specialists, geriatricians and geriatrics and advanced practice nursing fellows. PCP’s refer older adults with complex care needs through the EHR for virtual consultation by the ICT. Team members review medical records and call participants and caregivers to obtain permission for the consult, gather information on function, social factors, medical problems, and their perceived needs. The ICT meets to review each case and sends written recommendations to the PCP and patient. To date, the ICT performed consultations for 73 older adults with a mean age of 76 years. 69% were female. 71% were black and 26% white. Frequently identified needs included personal/home safety (74%), medication management (64.3%), food security (63.0%), cognition (49.3%), transportation (38.4%) and advance care planning (31.5%). In the 90 days before consultation, 32.9% of patients had ED visits and 21.9% were hospitalized. In the 90 days after, 24.7% had ED visits and 13.7% were hospitalized. (Differences were not statistically significant.) ICT provided virtual consultation for complex older adults with prevalent social needs and high rates of ED visits and hospitalization. The team worked with PCP’s to connect these patients more directly to community resources. Further study is needed to know rates of adherence with recommendations and true impact on health outcomes.

